# Hydatid Cyst of the Tibia

**DOI:** 10.1590/0037-8682-0082-2022

**Published:** 2022-04-29

**Authors:** Recep Tekin, Emin Özkul, Sait Anıl Ulus

**Affiliations:** 1 Dicle University, Faculty of Medicine, Department of Infectious Diseases and Clinical Microbiology, Diyarbakir, Turkey.; 2 Dicle University, Faculty of Medicine, Department of Trauma and Orthopedic Surgery, Diyarbakir, Turkey.

**FIGURE 1:**
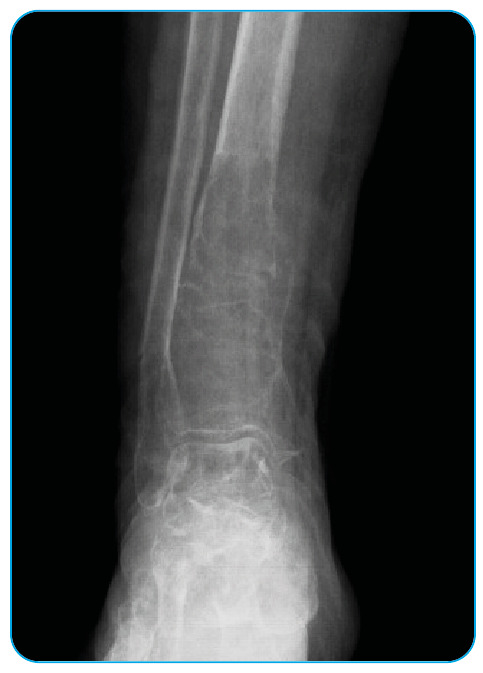
Radiological examination revealed multiple diffuse lytic areas with surrounding sclerosis distal to the tibia extending from the metaphysis to the diaphysis and pathological fracture.

**FIGURE 2:**
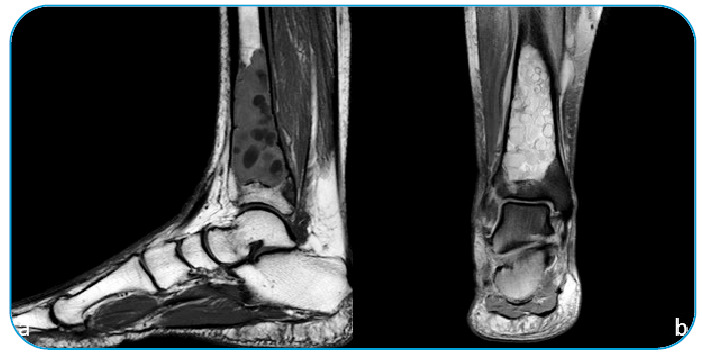
A sagittal **(a)** and coronal **(b)** slice of the MRI showing multicystic lesion in the distal tibia metaphysis.

**FIGURE 3:**
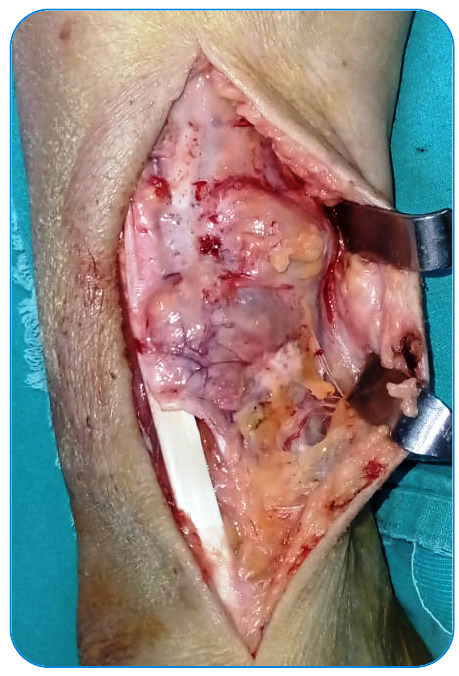
Intraoperative image.

A 63-year-old female patient had a 5-year history of limitation in the movement of her right leg and pain. She had dull aching pain in the leg with slight swelling. The physical examination of her leg revealed swelling and mildly painful movement with limitation. The radiological study revealed multiple diffuse lytic areas with surrounding sclerosis distal to the tibia extending from the metaphysis to the diaphysis and pathological fracture (Figure 1). Magnetic resonance imaging of the patient’s right leg revealed multiple, round, multivesicular T1 isointense and T2 hyperintense lesions in the distal tibia with cortical breach, and the extension into the adjacent soft tissue and right leg showed a pathological fracture of the distal tibia (Figure 2). Wide resection of the right distal tibia was performed, with care taken to maintain adequate margins of the healthy tissue around the associated soft tissue cystic components (Figure 3). The multiple daughter cysts were filled with a muddy substance, typical of hydatid disease. Histopathological examination confirmed the diagnosis of hydatid disease. The patient was treated with oral albendazole at 400 mg daily for 6 months. Pericystectomy combined with neoadjuvant therapy can help reduce the complications and recurrence in soft tissue hydatid cysts^1^ ^,^ ^2^. Osseous hydatidosis is an extremely rare disease, but it should be included in the differential diagnosis of patients with evidence of a destructive bone process, especially if they had a close contact with host animals or are emigrants from endemic countries^3^.
